# Preventing Dementia Using Saffron, The Kampo Medicine, Kamiuntanto, and Their Combination, Kamiuntantokabankoka

**DOI:** 10.3389/fphar.2021.779821

**Published:** 2022-03-04

**Authors:** Kenny Kuchta, Kosuke Aritake, Yoshihiro Urade, Nguyen Huu Tung, Chun-Su Yuan, Yui Sasaki, Koichi Shimizu, Yukihiro Shoyama

**Affiliations:** ^1^ Forschungsstelle für Fernöstliche Medizin, Department of Vegetation Analysis and Phytodiversity, Albrecht von Haller Institute of Plant Sciences, Georg August University, Göttingen, Germany; ^2^ Daiichi University of Pharmacy, Fukuoka, Japan; ^3^ Faculty of Pharmacy, Phenikaa University, Hanoi, Vietnam; ^4^ Department of Anesthesia and Critical Care, The University of Chicago, Chicago, IL, United States; ^5^ Association for Health Economics Research and Social Insurance and Welfare, Tokyo, Japan; ^6^ Faculty of Pharmacy, Nagasaki International University, Sasebo, Japan

**Keywords:** kamiuntanto, saffron, kamiuntantokabankoka, kampo (traditional Japanese herbal medicine), dementia prevention and control

## Abstract

The objective of this review is to evaluate the anti-dementia activities of saffron and its combination with Kampo medicine. The Kampo formula Kamiuntanto composed of 13 crude drugs is well known for its anti-dementia activity. A significant increase in choline acetyltransferase activity and mRNA levels were observed. *Polygala* radix was identified as the most essential component drug in Kamiuntanto, probably due to the saponins, tenuifolin, and sinapinic acid. Ginseng was also identified as an essential Kamiuntanto component in terms of its synergistic functions with *Polygala* radix. Saffron, which was recommended in the Bencao Gangmu for memory and dementia, and is used as an anti-spasmodic, anti-catarrhal, and sedative herbal drug. Saffron and its major constituent, crocin were shown to enhance learning-memory, non-rapid eye movement (rem) sleep, and inhibit depression and neuronal cell death due to strong anti-oxidant and anti-inflammation activities. In addition based on the epidemiological studies such as the treatment of sleeping disorders and the clinical trials of saffron for Alzheimer patients, we demonstrated the indirect and direct anti-dementia activities of crocin and saffron.

## Introduction

Globally, the incidence of neurodegenerative disorders such as dementia has increased with increased life expectancy. Previously, it was estimated there could be 81.1 million dementia patients by 2040 (Ferri et al., 2005). In Japan, 7.3 million cases are speculated by 2025, and by 2050, 10.2 million cases are expected (National Institute of Public Health, 2015). Consequently, speedy and rapid dementia innovations and prevention methods are required.

The Diagnostic and Statistical Manual of Mental Disorders (Battle, 2013) indicates that major dementia cases are classified as Alzheimer’s disease, vascular dementia, frontal lobe hyperthermia, dementia with Lewy bodies, Parkinson’s disease with dementia, Huntington’s disease with dementia, or a combination of the above. Of these conditions, Alzheimer’s disease is the most common, with aging the most important single risk factor. However, epidemiological studies have identified that lifestyle and eating habits influence the condition, suggesting that wine (Orgogozo et al., 1997) or fish (Kalmijn et al., 1997) reduce the probability of developing Alzheimer’s disease in old age. Similarly, Kampo medicine or other herbal remedies may also prevent dementia. Several studies have shown, via acetylcholine esterase inhibitor assays, that numerous pure compounds have anti-dementia activities (Ho et al., 2011; Natarajan et al., 2013), but no compounds have been clinically approved. In this review, phytochemicals such as Kamiuntanto and saffron which have anti-dementia activities are discussed.

### Anti-Dementia Active Compounds From Medicinal Plants

The alkaloid galantamine (brand name: Razadyne and GalantaMind™) was originally isolated from Galanthus nivalis L., but is now produced by chemical synthesis, and is a global anti-dementia drug for mild and moderate Alzheimer’s disease. Since 2016, Polygala tenuifolia Willd. root extract is also marketed as an over the counter (OTC) drug for memory improvement in Japan. Another similar product, Ginkgo biloba L. extract fraction (EGb761), which contains 24% flavonoid glycosides and 6% diterpene lactones, is an OTC drug for vascular dementia prevention in Europe (Clostre, 1999). Interestingly, Valeriana officinalis L. root, an OTC medicine for sleep disorders traditionally used in Europe, is reported to exert anti-dementia activity (Chen, 2016).

Of the many plants that exhibit pharmacological activities (e.g., acetylcholine esterase and monoamine oxidase inhibition) related to anti-dementia effects, anti-inflammatory activities, and learning and memory effects in animal models (Natarajan et al., 2013; Ho et al., 2011), the following are also commonly found in Kampo prescriptions: *Acorus gramineus* Aiton, *Angelica dahurica* (Hoffm.) Benth. & Hook.f.ex Franch. & Sav., *Aralia cordata* Thunb., *Codonopsis pilosula* (Franch.) Nannf., *Crocus sativus* L., *Curcuma longa* L., *Epimedium brevicornu* Maxim., *Gardenia jasminoides* J. Ellis, *Glycyrrhiza glabra* L., *Lycium chinense* Mill., *Magnolia officinalis* Rehder & E.H.Wilson, *Panax ginseng* C.A.Mey., *Perilla frutescens* (L.) Britton, *Polygala tenuifolia* Willd., *Zingiber offinale* Roscoe, *Rhodiola rosea* L., *Salvia miltiorrhiza* Bunge and *Uncaria rhynchophylla* (Miq.) Miq. (Li and Zhang, 2009).

### Kampo Formulas for Dementia

Kampo theory was originally based on Ancient Chinese Medicine (ACM) from the fifth and sixth centuries in China and Korea. ACM was primarily based on ancient empirical knowledge of diseases and treatments, that was collected in classics such as the Shānghán Lùn (傷寒論, Jap. Shokanron) and the Shénnóng Běn Cǎo Jīng (神農本草経, Jap. Shinnohonzokyo). ACM is the base of all later forms of East Asian Traditional Medicine such as Korean Medicine, contemporary Traditional Chinese Medicine (TCM), and Japanese Kampo Medicine. Currently, 148 Kampo formulas as finished pharmaceutical products (FPP) are covered by the Japanese National Health Insurance and widely used for many diseases. In traditional Kampo philosophy, dementia is caused by “Oketsu” (瘀血) or “blood stagnation,” which may be interpreted as circulatory disorders of the brain. Thus, therapies that enhance blood circulation are important for dementia therapy and prevention.

As indicated (Table 1), Kamiuntanto (Yabe et al., 1996; Yabe and Yamada, 1997a; Yabe and Yamada, 1997b; Yabe et al., 1997), Tokishakuyakusan (Kim and Cho, 2020), Yokukansanchinpihange (Okamoto, 2017), Hachimijiogan (Iwasaki et al., 2004), Chotosan (Terasawa et al., 1997), and Orengedokuto (Fujiwara et al., 2018) have been used for dementia therapy and/or prevention in Japan. Of these medicines, Kamiuntanto has been the most studied.

**TABLE 1:** Clinically used anti-dementia Kampo formulae in Japan.

Type of dementia	Kampo prescription	Raw drugs in kampo formulas (scientific names are incorporated from Japanese pharmacopoeia)	References
Alzheimer’s disease	Kamiuntanto (加味温胆湯)	*Pinellia ternata* Breitenbach (Araceae), *Polygala tenuifolia* Willd. (Polygalaceae), *Zizyphus jujuba* Miller var. *spinosa* (Bunge) Hu ex H. F. Chou (Rhamnaceae), Processed *Rhemania glutinosa* Liboschitz (Scrophulariaceae), *Panax ginseng* C.A. Meyer (Alariaceae), *Z. jujuba* var. *inermis* Rehder (Rhamnaceae), *Bambusa tuldoides* Munro (Gramineae), *Poria cocos* Wolf (Polyporaceae), Immature *Citrus aurantium* Linn. var. *daidai* Makio or *C. natsudaidai* Hayata (Rutaceae), *C. unshiu* Markovich or *C. reticulata* Blanco (Rutaceae), *Glycyrrhiza glabra* Linn. or *G. uralensis* Fisher (Leguminosae), *Zingiber offinale* Roscoe (Zingiberaceae), *Scrophularia ningpoensis* Hemsl. (Scrophulariaceae)	Yabe et al. (1997)
			Yabe and Yamada (1997b)
			Yabe and Yamada (1997a)
			Yabe et al. (1996)
Alzheimer’s disease	Tokishakuyakusan (当帰芍薬散)	*Angelica acutiloba* (Siebold and Zucc.) Kitag. (Umbelliferae), *Cnidium offcinale* Makino (Umbelliferae), *Paeonia lactiflora* Pallas (Paeoniaceae), *Atractylodes lancea* De Candolle or *A. chinensis* Koidzumi (Compositae), *Alisma oriental*e Juzepczuk (Alismataceae)	Kim and Cho (2020)
Vascular dementia	Chotosan (釣藤散)	*Uncaria rhynchophyla* Miquel. or *U. macophylla* Wallich (Rubiacerae), *P. ginseng* C.A. Meyer (Alariaceae), *P. cocos* Wolf (Polyporaceae), *P. ternata* Breitenbach (Araceae), *Ophiopogon japonicus* Ker-Gawler (Liliaceae), *C. unshiu* Markovich (Rutaceae), *Saposhnikovia divaricata* Schischkin (Umlelliferae), *G. glabra* Linn. or *G. uralensis* Fisher (Leguminosae), *Gypsum*, *Z. offinale* Roscoe (Zingiberaceae), *Chrysanthemum morifolium* Ramatulle or *C. indicum* Linn. (Compositae)	Terasawa et al. (1997)
Vascular dementia	Yokukansanchinpihange (抑肝散加陳皮半夏)	*A. lancea* De Candolle (Compositae), *P. cocos* Wolf (Polyporaceae), *A. acutiloba* (Siebold and Zucc.) Kitag. (Umbelliferae), *C. offcinale* Makino (Umbelliferae), *U. rhynchophyla* Miquel. or *U. macophylla* Wallich (Rubiacerae), *Bupleurum falcatum* Linn. (Umbelliferae), *G. glabra* Linn. or *G. uralensis* Fisher (Leguminosae), *P. ternata* Breitenbach (Araceae), *C. unshiu* Markovich or *C. reticulata* Blanco (Rutaceae)	Okamoto (2017)
Vascular dementia	Orengedokuto (黄連解毒湯)	*Coptis japonica* Makino or *C. chinensis* Franchet (Ranunculaceae), *Phellodendron amurense* Ruprecht or *P. chinense* Schneider (Rutaceae), *Gardenia jasminoides* J.Ellis, *Scutellaria baicalensis* Georgi (Labiatae)	Fujiwara et al. (2018)
Mix of the above	Hachimijiogan (八味地黄丸)	*R. glutinosa* Liboschitz (Scrophulariaceae), *Cornus officinalis* Siebold et Zuccarini (Cornaceae), *Dioscorea japonica* Thunberg or *D. batatas* Decaisne (Dioscoreaceae), *P. cocos* Wolf (Polyporaceae), *A. oriental*e Juzepczuk (Alismataceae), *P. lactiflora* Pallas (Paeoniaceae), *Cinnamon cassia* Blume (Lauraceae), *Aconitum japonicum* Thunberg or *A. carmichaeli* Debeaux (Ranunculaceae)	Iwasaki et al. (2004)

### Kamiuntanto

Kamiuntanto is traditionally used for neurosis, insomnia, gastroptosis, gastroparesis, and weakness after a major illness. Kamiuntanto formula (Table 1) (Yabe et al., 1996; Yabe and Yamada, 1997a; Yabe and Yamada, 1997b; Yabe et al., 1997) enhances nerve growth factor (NGF) secretion and choline acetyltransferase (ChAT) activity. In a cell culture model of rat embryo basal forebrain cells cultured in medium containing Kamiuntanto extract, a significant increase in ChAT activity and mRNA levels was recorded (Yamada et al., 1997; Yabe and Yamada, 1997a). Furthermore, Kamiuntanto ameliorated cholinergic shortages in aging rats (Yabe et al., 1996) and subsequent investigations reported that cAMP and c-fos mRNA were closely related to NGF biosynthesis (Yabe and Yamada, 1997b). To make clear the mechanism for the memory improving activity of Kamiuntanto, Hong et al. confirmed the expressions of protein kinase B, cAMP response element-binding protein, brain-derived neurotrophic factor and doublecortin in the hippocampal CA1 and dentate gyrus regions in mice by immunohistochemical and blotting techniques (Hong et al., 2011). Interestingly, while a variant extract of Kamiuntanto without *Polygala* radix displayed no increased ChAT activity, an oral administration of *Polygala* radix extract alone induced ChAT activity. Subsequently, onjisaponin and sinapinic acid were independently tested and generated the same results as Kamiuntanto formulas (Yabe et al., 1997). In studies, the 13 Kamiuntanto components were individually removed so the effects of the remaining 12 could be assayed. This methodology is similar to knockout extract strategies which remove a target compound (antigen) from crude extracts using one step immunoaffinity separation techniques (Shoyama, 2011; Uto et al., 2012; Hsu et al., 2020).

The *P. tenuifolia* Willd. constituent, tenuifolin also inhibited β-amyloid secretion both *in vivo* and *in vitro* (Lv et al., 2009). Furthermore, tenogenic reduced antibody production by inhibiting β-secretase activity (Jia et al., 2004). Recently, multiple neuroprotective effects induced by *P. tenuifolia* Willd. were reported as a potential preventative therapy for Alzheimer’s disease (Deng et al., 2020). Thus, since 2016, *P. tenuifolia* Willd. root extract has been marketed as an OTC drug for memory preservation in Japan.

Approximately 2000 years ago, *P. ginseng* C.A.Meyer, *P. tenuifolia* Willd., *A. gramineus* Aiton, and *P. cocos* Wolf were listed in the Shennong Ben Cao Jing (神農本草経) as having memory enhancement and related psychological effects. Among the characteristic constituents of ginseng, ginsenoside was shown to exhibit a wide pharmacological activity spectrum, including analgesic, cholesterol biosynthesis and neural lipid synthesis, and adrenal cortex hormone enhancing activities. Furthermore, it improves memory and learning, central nervous system excitation, and promotes DNA and RNA synthesis (Leung and Wong, 2010). Ginseng also promotes neuronal cell growth and survival, and was shown to rescue neuronal cell death both *in vivo* and *in vitro* (Lee et al., 2001). Ginseng increases *in vivo* choline acetyltransferase levels suggesting that ginsenosides strengthen central cholinergic functions, and could be used to treat dementia (Rudakewich et al., 2001). Furthermore, Yamaguchi et al. reported that ginsenosides enhanced learning and memory performances in both brain damaged and/or aging mice (Yamaguchi et al., 1996). Itoh et al. reported that ginsenosides activated norepinephirine and dopamine in the cerebral cortex, to facilitate increased attention, processing cognition, and motor function activation (Itoh et al., 1989). Ginsenoside Rg1 increased neuronal precursors and was mechanically important in terms of its anti-aging activity effects, such as learning and memory (Shen and Zhang, 2003). Zhang et al. demonstrated that the anti-apoptosis activity of ginsenoside-Rg2 counteracted vascular dementia in an *in vivo* animal model (Zhang et al., 2008). Huang et al. showed that ginsenoside Rc activated SIRT1, which protected neurons from mitochondrial damage (Huang et al., 2021). Hence, not only *P*. *tenuifolia*, but also ginseng and its constituents displayed anti-dementia activities.

Ginseng, ginsenoside, and ginsenoside Rg3 also increased the postoperative life span of patients with non-small cell lung cancer (Lu et al., 2008). Within this therapeutic context, Yang et al. reviewed 257 dammarane-type ginsenosides from numerous *Panax* species, many of which showed promising pharmacological activities (Yang et al., 2014). For example, since ginsenoside Rc has anti-dementia activity (see above) but its concentration as determined by eastern blotting fingerprint for various *Panax* spp. (Sasaki et al., 2021) is quite low, it might be interesting to develop a method to induce the transformation of other minor ginsenosides into ginsenoside Rg3 in order to develop antidementia drugs.

We discussed the anti-dementia activities of two major crude drugs in the Kamiuntanto prescription, *P. tenuifolia* Willd. and *P. ginseng* C.A. Meyer previously. It became evident that the other crude drugs prescribed in Kamiuntanto showed anti-dementia activities as follows. *G. glabra* Linn. (Licorice root) is known to promote light movements, extend life span, and improve both physical and mental health as described in the Shennong Bao Jing. The pharmacological activities of licorice root can also improve mental function in patients with dementia, including AD. In fact aqueous extract of licorice root enhanced learning and memory in different type of models. Among its components, glycyrrhizin is used as a therapeutic drug for the treatment of liver disease and allergies in Japan. Regarding dementia Soo et al. investigated that glycyrrhizin significantly attenuated mitochondria-mediated cell death and decrease of glutathione due to neurotoxin, 1-methyl-4-phenylpyridinium resulting in the protective effect of glycyrrhizin on mitochondrial damage and cell death in PC-12 cells associated with dementia (Yim et al., 2007). The constituents of licorice root, especially flavones and isoflavones such as liquiritin, isoliquiritin, and coumestrol, are naturally occurring bioactive compounds although the ability of flavonoid glycosides to pass through the blood brain barrier (BBB) and reach the central nervous system is unclear. Therefore, several flavonoid glycosides and aglycones in *Glycyrrhiza* species root might be developed as functional compounds for the treatment of dementia. Liquiritigenin shows a selective estrogen receptor-β which are distributed in the brain centers of learning and memory, agonist (Mersereau et al., 2008). Further, liquiritigenin has neuroprotective activity against β-amyloid peptide (Aβ) in rat hippocampal neurons indicating that the pretreated neurons with liquiritigenin in the presence of Aβ increased cell viability and the treatment decreases Aβ-induced intracellular Ca^2+^ concentration and ROS level resulting in the decrease of apoptotic rate (Liu et al., 2009). Regarding *P. cocos* Smriga et al. showed that a single oral administration of *P. cocos* significantly intensified the formation of long-term potentiation (LTP) which is deeply involved to memory in the dentall gyrus (Smriga et al., 1995). Ban et al. reported that the rat cortical neurons pretreated by young *Phyllostachys nigra* (Bambu tree) methanol extracts were protected from Aβ-induced increase of cytosolic calcium concentration resulting that *P. nigra* prevents Aβ-induced neuronal cell damage *in vitro* (Ban et al., 2005). Pretreatment with *C. unshu* immature peel and its component, nobiletin inhibited individually cell death due to hydrogen peroxide induced the expression of phospho-Jun N-terminal kinases and p-p38 proteins in HT22 cells although the peel and nobiletin suppressed p-JNK and p-p38 without changing JNK or p38. These evidences confirm that the peel and nobiletin can protect against hydrogen peroxide-induced cell death in HT22 neurons *via* mitogen-activated protein kinases and apoptotic pathways (Cho et al., 2015). When rats were injected with Aβ_1-40_ into the hippocampus, the ability of spatial learning and memory decreased. However, the treatment with harpagoside, a constituent of *Scrophularia ningpoensis* improved Aβ_1-40_-induced behavioral damage (Li et al., 2015). The human monocytic cell line resemble human microglial cells (THP-1 cells) was incubated with ginger extract or with LPS, TNF-α, IL-1β or Aβ-protein resulted that the addition of ginger extract prohibited the expression of TNF-α, IL-1β, COX-2 and MCP-1. From this result the ginger extract could be used for delaying the onset and the progression of neurodegenerative disorders (Wang et al., 2015). When the methanol extract of dry ginger was tested for DPPH assay and FRAP assay, respectively to show the antioxidant activity and the Ellman’s assay for the extract indicated the cholinesterase inhibition. Furthermore, the extract ameliorated the cell survival for Aβ-induced toxicity in primary rat hippocampal cell culture. This result together with the above evaluation suggested that the extract of dry ginger is effective for Alzheimer’s disease (Mathew and Subramanian, 2014). The extract of steamed *R. glutinosa* Liboschitz root was investigated daily dose for rats injected scopolamine before 1 h for 14 days. The results were evaluated by a passive avoidance test and the Morris water maze test, and the activities of choline acetyltransferase and acetylcholinesterase in the hippocampus. The extract improved memory dysfunction behaviorally and cholinergicaly resulted that the extract could be used to improve cognitive function by activation of cholinergic enzyme (Lee et al., 2011).

The effect of spinosin isolated from the seeds of *Z. jujuba* Miller var. *spinosa* (Bunge) Hu ex H. F. was investigated on cholinergic induced memory dysfunction and behavioral task using the passive avoidance, Y-maze, and Morris water maze tasks. Spinosin significantly improved scopolamine-induced cognitive performance on behavioral tasks. In order to confirm the mechanism for improving activity of spinosyn, the survey of receptor antagonism and Western blotting were examined resulted that the improving effect of spinosin on scopolamine-induced memory impairment was significantly antagonized by 8-hydroxy-2-(di-N-propylamino) tetralin, a 5-HT1A receptor agonist and spinosin significantly increased the expression levels of phosphorylated extracellular signal-regulated kinases and cAMP response element-binding proteins in the hippocampus. From these results it became clear that the memory-improving activity of spinosin might be depend on the serotonergic neurotransmitter system. Therefore, spinosin and/or *Z. jujuba* Miller var. *spinosa* (Bunge) Hu ex H. F. could be applied for cognitive dysfunction like Alzheimer’s disease (Jung et al., 2014). The crude drugs except *Pinellia ternate* and *Citrus aurantium* prescribed in Kamiuntanto formula have been surveyed their pharmacological activities regarding cognitive performance. They all indicated the activities for solving cognitive problem. So that their synergistic effects might be important for the documented effects of the Kamiuntanto formula.

### Saffron Pharmacological Activities

**FIGURE 1:**
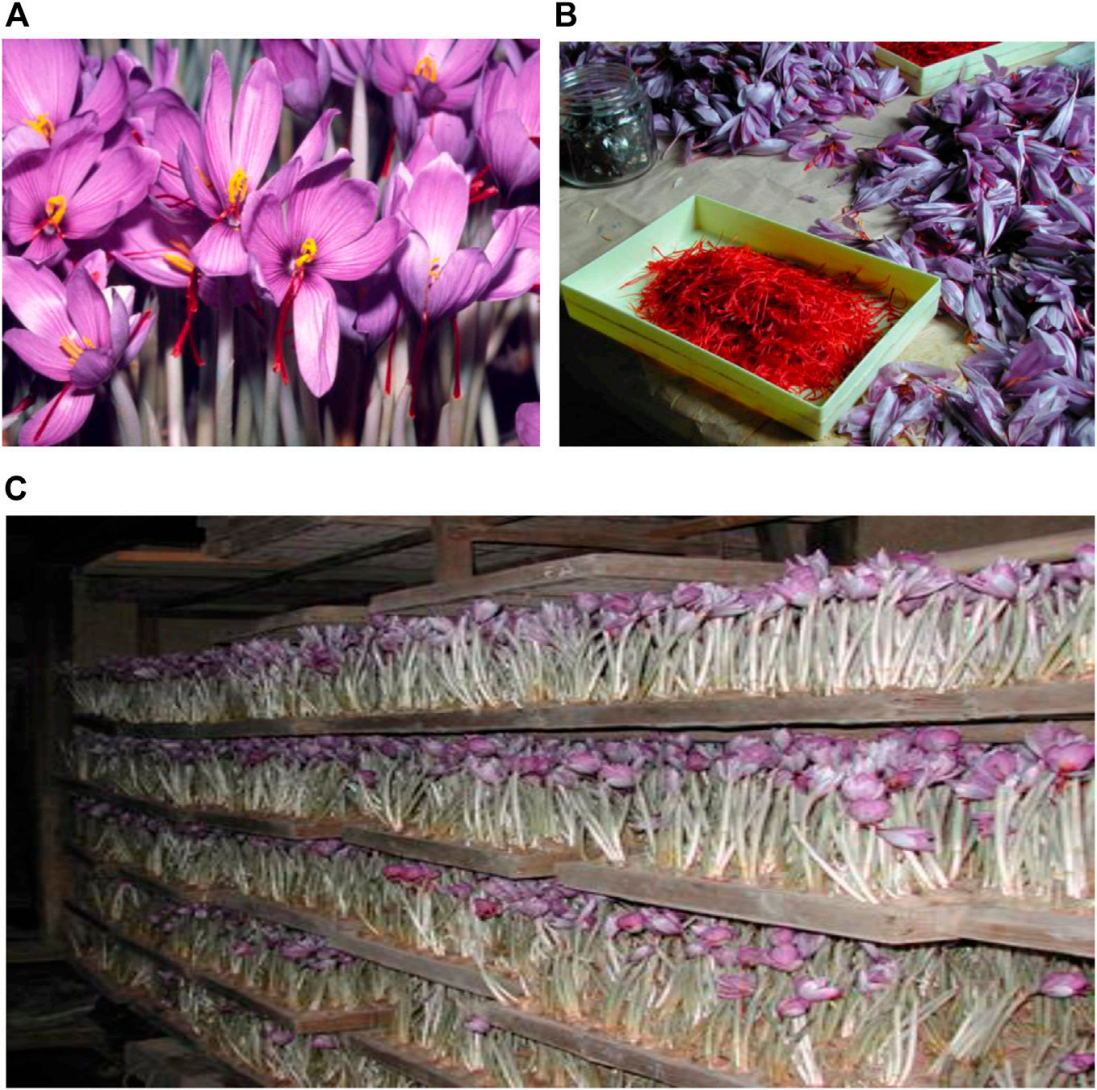
*Crocus sativus* L. **(A)** and saffron **(B)**. Indoor crocus cultivation in Oita-ken, Japan **(C)**.

*Crocus sativus* L. (Iridaceae; a perennial herb) was documented as cultivated on Crete approximately 3,500 years ago, but today, it is widely cultivated in Iran, Greece, Spain, Morocco, and domestically in Japan, for its red stigmata and saffron (Figure 1).

As far back as 1578 AD, saffron was identified in the Bencao Gangmu (本草綱目) for its neurological functions on memory and dementia. Saffron is known for its anti-convulsion, sedative, and heart-blood disorder qualities (Rigobello et al., 2002; Lee et al., 2005; Aung et al., 2007). The major constituent, crocin was shown to exert anti-cancer (Aung et al., 2007; Chryssanthi et al., 2007), anti-hypolipidemic (Lee et al., 2005; Sheng et al., 2006), anti-atherosclerotic (Xu et al., 2005; Xu et al., 2006; Xu et al., 2009), and anti-inflammatory effects (Ochiai et al., 2007; Naghizadeh et al., 2008). Crocin’s neuro-protective activities related to dementia were investigated using cerebral ischemia, (Papandreou et al., 2006), memory impairment (Sugiura et al., 1994; Sugiura et al., 1995a), and N-methyl-D-aspartate receptor (NMDA) malfunction models (Abe et al., 1998).

The medical used of imported saffron in Japan has been documented since the start of the Edo-Bakufu in the early 1600s. In 1886, the first Japanese Pharmacopoeia accepted saffron as a non-prescription drug, a status that has remained unchanged to the present. Between 1830 and 1844, domestic saffron cultivation commenced in Oita-ken in western Japan (Figure 1).

The blooming period of the crocus is once a year and the stigma harvest time is very short; therefore, saffron prices are very high when compared with other herbal medicines (Morimoto et al., 1994). Moreover, saffron quality depends on weather conditions, however to alleviate this, indoor cultivation systems were developed in Japan in the early 20th century (1910) (Figure 1). Under these conditions, approximately 90,000–100,000 flowers generate 5.0 kg fresh saffron, in turn generating 1.0 kg dried drug. Indoor cultivation systems facilitate the easy collection of saffron adjusting most suitable full blooming season, therefore indoor cultivation is less labor intensive and advantageous for quality control measures (Morimoto et al., 1994).

**FIGURE 2:**
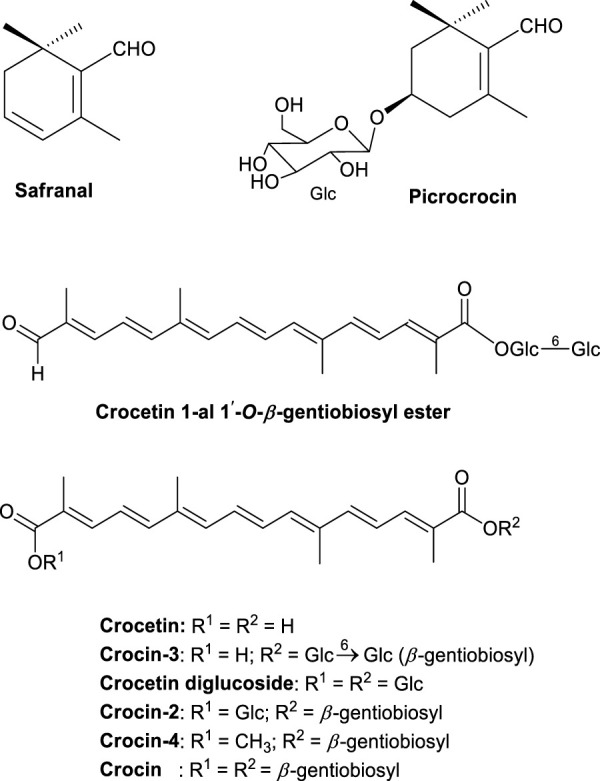
The major saffron phytochemical constituents.

The dominant components of saffron are carotenoids, picrocrocin, and safranal (Figure 2). Recently, the novel crocetin glycoside trans-crocetin-1-al 1-O-β-gentiobiosyl ester was isolated in our laboratory (Tung and Shoyama, 2013). Drying saffron should be completed within 30–45 min; β-glucosidase remains active as long as moisture is contained in the plant material and may destroy the typical ester glycoside conjugation of carotenoid pigments, e.g., crocetin-diglucoside, -2, -3, -4 and crocetin di-(β-D-digentiobiosyl)-ester. The dried saffron is then chilled and preserved free from moisture, as β-glucosidase remains active under moisture conditions thereby causing hydrolysis (Morimoto et al., 1994). High performance liquid chromatography (Morimoto et al., 1994) and monoclonal antibody (MAb) (Xuan et al., 1999) technologies are commonly used for saffron quality control. Crocin levels in official saffron extracts are approximately 30% higher than other components. Crocin is a major contributor to the pharmacological activity of saffron as identified by extract fractionation bioactivity assays (Morimoto et al., 1994).

Saffron may be used as an anti-spasmodic and anti-catarrhal therapy in neuronal and heart-blood disorders (Rigobello et al., 2002; Lee et al., 2005; Aung et al., 2007). Crocin exerts anti-oxidant (Rigobello et al., 2002; Ochiai et al., 2004; Lee et al., 2005), anti-cancer (Aung et al., 2007; Chryssanthi et al., 2007), hypolipidemic (Lee et al., 2005; Sheng et al., 2006), anti-atherosclerotic (Xu et al., 2005; Xu et al., 2006; Xu et al., 2009), and anti-inflammatory effects (Ochiai et al., 2007; Naghizadeh et al., 2008). Brain dysfunction models performed the protection of neuronal function such as cerebral ischemia (Papandreou et al., 2006), Alzheimer’s disease (Lechtenberg et al., 2008), NMDA receptor (Abe et al., 1998), and memory impairment (Sugiura et al., 1994; Sugiura et al., 1995a), which are closely related to dementia. It is accepted that learning and memory processes via long-term potentiation (LTP) occur in the hippocampus (Abe et al., 1991).

Previously, several studies investigated saffron extracts and crocin in mice with learning behaviors (Zhang et al., 1994; Sugiura et al., 1995b; Dashtira et al., 2009; Akbari et al., 2019; Roustazade et al., 2021), and LTP in the CA1 region of the rat hippocampus (Zhang et al., 1994; Sugiura et al., 1995b).

Crowe et al. reported that programmed cell death (apoptosis) in neurons occurred in the brains stripped of oxygen by stroke (Crowe et al., 1997), trauma (Hill et al., 1995), and patients with Alzheimer’s disease (Pettmann and Henderson, 1998). Although the value of this approach is not yet proven beyond reasonable doubt, the prevention of neuronal apoptosis could become a therapeutic strategy for neurodegenerative disease.

### Learning and Memory Activities Induced by Saffron and Crocin

We observed that mice with saffron extract and the control group on memory and learning behavior were nothing of differences supporting no effect of saffron for healthy control mice but improving potentiality time induced by ethanol (Sugiura et al., 1995b). Saffron extracts prevented impairments in memory induced by ethanol in passive avoidance studies (Zhang et al., 1994). Saffron also dose-dependently and significantly ameliorated increased memory errors induced by ethanol (Sugiura et al., 1995b). As an active constituent in saffron extracts it is easily suggested to be crocin because higher concentration. In fact, approximately 15% of crocin is contained in fresh dried saffron and results in 30% crocin levels in ethanol extracts (Morimoto et al., 1994). The activity of saffron extract appeared from 125 mg/kg which contained nearly 40 mg of crocin and dose-dependently increased (Sugiura et al., 1995b). Further studies have indicated that the oral administration of at least 50 mg/kg crocin improved impaired memory induced by ethanol (Sugiura et al., 1995c). Thus, crocin is a major active component of saffron and exerts similar pharmacological effects as saffron extract, although its activity is slightly different (Sugiura et al., 1995c).

### Saffron and Crocin Activities Against LTP

In LTP studies, the intracerebroventricular injection of saffron extract dose-dependently decreased the negative effects of ethanol on LTP (Sugiura et al., 1995a; Sugiura et al., 1994). A crocin injection (50 mg/kg) at 5 min before ethanol treatment exhibited 84% LTP when compared with controls. As previously discussed, several crocetin glucose esters were isolated, and included crocin with 2-gentiobiose ester, crocetin gentiobiose glucose ester, and crocetin di-glucose ester (Figure 2) in molecules. When compared with the LTP blocking effects of ethanol the others rather than crocin were lower resulting that the improvement effect against blocking by ethanol was relatively reflected to the sugar number. This tendency was reported that the saponin’s haemolytic activity of di- and triglycoside saponins were higher than that of monoglycoside (Voutquenne et al., 2002). Also, the hemolytic activity of saikosaponins was dependent on sugar numbers (Abe et al., 1978). From these observations, crocin appears to be the major active component in saffron in terms of its impact on learning and memory.

### Crocin Activity on Pheochromocytoma Cell Death Induced by Serum/Glucose Deprivation in PC-12 Cells

To confirm crocin incorporation on PC-12 cells, a MAb against crocin was generated for immunostaining (Xuan et al., 1999). The method confirmed crocin incorporation into PC-12 cells when compared with control cells (Ochiai et al., 2004).

When cells were cultured in Dulbecco’s modified Eagle’s medium (DMEM) containing glucose and serum (DMEM+), a normal morphology was observed after 24 h culture. However, cells cultured in serum- and glucose-free medium (DMEM-) for 24 h were rounded, causing a necrotic or apoptotic morphology and 60% cell death. However, this was recovered to 85% cell survival by crocin (10 μM) addition to DMEM-, and was reportedly *via* TNF-α inhibition, in a dose-dependent manner (Soeda et al., 2001). However, it is accepted that serum (Batistatou and Green, 1991; Oppenheim, 1991; Rukenstein et al., 1991) or NGF (Mesner et al., 1992; Pittman et al., 1993) removal induces apoptosis in PC-12 cells. It was reported that serum elimination from culture medium increased intracellular ceramide levels in undifferentiated HN9.10e cells and induced apoptosis (Colombaioni et al., 2002). In fact, when PC-12 cells were cultured for 3 h in DMEM-media, ceramide levels increased 3.5-fold when compared with DMEM+ conditions. Although fumonisin B1 (FB1) inhibits the *de novo* synthesis of ceramide at 10–30 μM (Wang et al., 1991; Merrill et al., 1993), FB1 exerted no decrease in ceramide levels. This phenomenon may have occurred via the combinatorial function of sphingomyelin (SM) and SAPK/JNK signaling pathways in the stress-induced apoptosis of U937 and BAE cells (Verheij et al., 1996). However, this hypothesis requires further investigation.

### The Anti-apoptotic Activities of Crocin

Previously, PC-12 cells in DMEM-media displayed morphological changes and membrane peroxidation leading to decreased superoxide dismutase (SOD) activity (Ochiai et al., 2004). Annexin V is typically used to stain phosphatidylserine (PS) lipids in peroxidized membrane lipids. While PS lipids are usually fixed to inner membranes, they become morphologically altered and bind to outer membranes under oxidative stress. PS externalized membranes are detected by annexin V as ring-like staining reflective of apoptotic activity. PC-12 cells cultured in DMEM-for 6 h exhibited 1.8-fold increased peroxidized membrane lipid levels, whereas SOD activity had decreased to 14% when compared with control cells in DMEM+.

To confirm the anti-oxidant activity of crocin, peroxidized membrane lipids and restored SOD activity in PC-12 cells in DMEM-plus crocin were analyzed and compared to α-tocopherol as a positive control. Crocin significantly weakened peroxidized membrane lipid formation and preserved SOD activity when compared with α-tocopherol treated cells (Soeda et al., 2001).

### The Effects of Crocin on Neural Sphingomyelinase in PC-12 Cells

PC-12 cell homogenate supernatants after substituting the reaction medium for 50 mM sodium acetate buffer (pH 5.6) were investigated to search the activity of magnesium-dependent neural sphingomyelinase for the determination of origin of accumulated ceramide. It became evident that the activity in DMEM-cells reached a maximum 1 h culturing and 3 h late backed to the revel of control cells without the time dependent change. This phenomenon indicated no effects of serum and glucose withholding for 3 h. In contrast, crocin supplementation to the medium dose-dependently inhibited enzyme activities at 1 and 2 h of culturing. When 1 or 10 μM crocin was added to DMEM-medium and cultured for 2 h, no inhibition of neural sphingomyelinase activity occurred in this medium. Previously, we had indicated that GSH functioned as a physiological inhibitor of magnesium-dependent neural sphingomyelinase in plasma membranes (Ochiai et al., 2004), 1 and 10 mM GSH added in the medium inhibited the enzyme activity dose-dependently.

### Increased Intracellular GSH Levels in Serum and Glucose Deprived PC-12 Cells

We investigated intracellular GSH levels in serum and glucose deprived PC-12 cells and observed that GSH levels 3 h decreased by 50% when compared with control cells, and recovering to the constant revel. In contrast, 10 μM crocin significantly boosted intracellular GSH levels and maintained high levels to inactivate neural sphingomyelinase. To make sure the mechanism of increasing GSH level by crocin, the addition of 10 μM crocin in PC-12 cells cultured in DMEM-induced the higher GC activity time-dependently although the GR activity in PC-12 cells in EMEM-decreasing time-dependently. Nakajima et al. (2002) suggested that NGF increased c-GCS activity at the transcription level and extended the half-life of c-GCS mRNA. Urata et al. (1996) indicated that GSH synthesis was regulated by c-GCS, whose activity was related to increased TNF-α or interleukin (IL)-1β in mouse endothelial cells, and further related to mRNA expression. IL-6 also stimulated c-GCS mRNA expression and increased enzyme activity resulting in increased GSH levels in PC-12 cells. These data showed that crocin had no significant effects on GPx activity in cells. Ten μM crocin increased the c-GCS mRNA expression twice in PC-12 cells cultured in DMEM-inducing the higher enzyme activity in the cells although the mRNA levels in the control PC-12 cells did not increase. From this evidences, crocin increased GSH levels in PC-12 cells in DMEM-media, resulting in survival against the PC-12 cell death.

### Non-Rapid Eye Movement Sleeping Effects of Crocin

**FIGURE 3:**
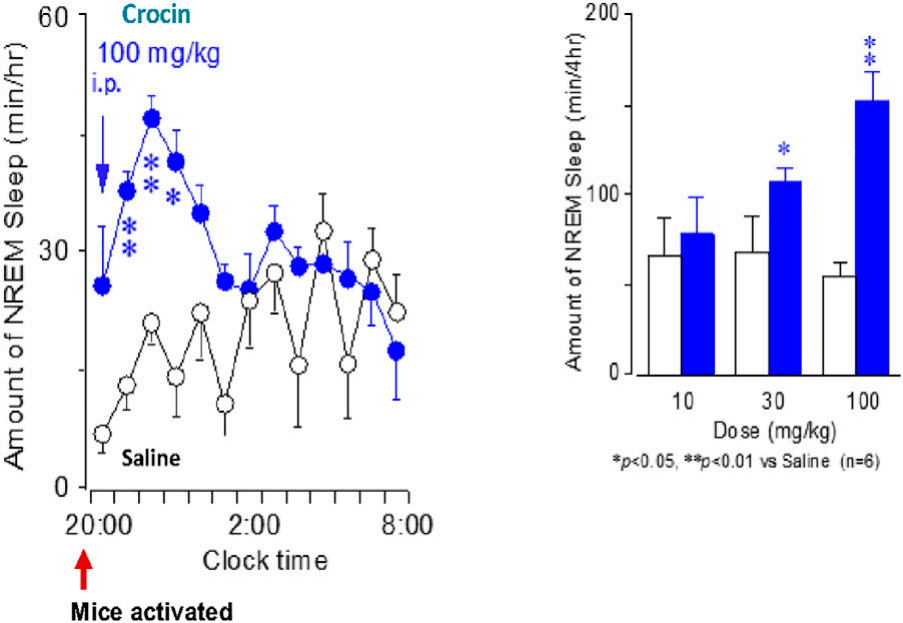
The effect of crocin on sleep architecture in a mouse model.

Saffron and Kampo medicines promoted sleep during mental disorder therapy. The combination of saffron and Saikokaryukotsuboreito or Sansoninto was previously used as a sleep promoter in Japan (Matsuhashi, 1993). Therefore, we investigated the sleeping efficacy of crocin in mice after intraperitoneal administration with crocin at 20:00 in the evening. Wakefulness, non-REM sleep, and REM sleep after prescribed crocin (100 mg/kg) or vehicle were compared (Figure 3). Non-REM sleep occurred due to 100 mg/kg crocin administration and the intensity increased immediately after injection and the efficacy was statistically significant during 4 h after the injection. The extension of non-REM sleeping times was induced by the reduction of wake continuing 4 h after injection. However, crocin did not affect REM sleep. In contrast, mice treated with the vehicle were awake for longer between 20:00 and 01:00. From these data, crocin induced non-REM sleep with no typical side effects, such as rebound insomnia. When a 30 mg/kg crocin injection was administered, the same evidence occurred with shorter non-REM sleeping term for 1–2 h after the injection. The total time of non-REM and REM sleep and wakefulness in the 4 h period after crocin injection was calculated; crocin at 10 mg/kg had no effect on the cumulative amount of non-REM and REM sleep and wakefulness at 4 h after injection. However, 30 and 100 mg/kg crocin injections significantly increased total non-REM sleep to 160 and 270%, respectively, and significantly decreased total wakefulness to 20 and 50%, respectively, without changing REM sleep levels during 4 h as compared with the control (Masaki et al., 2012).

### Clinical Trials on the Effects of Saffron on Sleep Architecture

To confirm increased sleep quality during saffron therapy, a double-blinded clinical trial using 21 healthy adults randomly assigned to either a saffron extract group (0.6 mg/day) or a placebo group was conducted. The trial demonstrated a significant reduction in Pittsburgh Sleep Quality Index scores in the saffron group. Furthermore, a significant positive effect of the saffron extract on daytime dysfunction appeared in the extract group when compared with the placebo group (Nishida et al., 2018).

### The Relationship Between Sleep and Dementia

Recent evidence has suggested that sleep disturbance may lead to increased inflammatory processes, which in turn may lead to Alzheimer’s disease (Irwin and Vitiello, 2019). Several meta-analyses and systematic reviews indicated that sleep disturbance may be an important risk factor, and thus an important target for Alzheimer’s disease prevention (Bubu et al., 2017; Shi et al., 2018; Livingston et al., 2020). Furthermore, the Hisayama epidemiological study in Fukuoka, Japan provided clear evidence that sleep disorders and the concomitant use of hypnotic drugs resulted in an increased risk of dementia in elderly patients. When patients with daily sleep durations shorter than 5.0 h were compared with those reporting more than 10 h, the risk of dementia, such as vascular dementia and Alzheimer’s disease for the short duration cohort, was increased 2-fold when compared with the 10 h cohort. To explain this, poor sleep quality may induce brain aging and lead to β-amyloid accumulation and Alzheimer’s disease. Alternatively, sleep disturbance may promote inflammation and induce dementia and depression (Ohara et al., 2018). As saffron and crocin improve sleep quality (Masaki et al., 2012), this sleep duration extension may critically decrease the risk of dementia. A recent survey reported a connection between sleep disorders and dementia (Livingston et al., 2020). In our saffron research, we observed a 3-fold stronger anti-oxidant activity for crocin when compared with α-tocopherol (Ochiai et al., 2004). We further showed that previously proven crocin activity against colorectal cancer in mice was based on its strong anti-inflammatory activities (Kawabata et al., 2012). These observations suggested that the anti-dementia activities of saffron may be based on the same anti-inflammatory activities in mice. Lastly, crocin also exerted direct effects on hippocampal neurons via the NMDA receptor (Abe et al., 1998) and may therefore exert direct anti-dementia activities.

In Japan, combinations of classical Kampo formulas with saffron have been used in several clinical disciplines such as obstetrics and gynecology, psychiatry, and cardiology. For example, saffron (100 mg–1 g/day) was clinically used for patients with sleep disorders, together with Kampo formulas such as Saikokaryukotsuboreito, Hangekobokuto, Sansoninto, Daijokito, and Chotosan (Matsuhashi, 1993) or combinations thereof. Therefore, these Kampo-saffron combinations offer new and interesting avenues to develop future anti-dementia therapies.

### Saffron and Crocin Activities for Anti-Depression Therapy

The neuroprotective activities of crocin were investigated using several brain disorder models, such as cerebral ischemia (Ochiai et al., 2007), Alzheimer’s disease (Akhondzadeh et al., 2010a), depression (Hausenblas et al., 2013), and memory impairment (Sugiura et al., 1994; Sugiura et al., 1995c) which are all closely related to dementia. Depression—depending on the specific variant and physiological mechanism—may be closely related to early stage dementia. Depression was identified as a risk factor for dementia after 2–17 years of meta-analysis (Prince et al., 2014). In the Whitehall Study in the United Kingdom, in a follow-up of 10,189 patients, depression increased dementia risk later in life (Almeida et al., 2017). A 14-years study, including 4,922 initially cognitive healthy men of 71–89 years old, reported that depression induced a significant incidence of dementia (Kelly et al., 2017).

Since sleeping disorders and depression are closely associated with dementia, the indirect relationship between saffron and crocin and dementia via sleeping disorders or depression was correctly determined.

### Saffron in Anti-dementia Therapy

A double-blinded, phase II study on 55 year old or older Alzheimer’s patients (54 patients) was performed over 22 weeks. Patients randomly received a 30 mg saffron capsule/day or 10 mg donepezil/day as a positive control. Saffron showed almost similar efficacy as donepezil in mild to moderate Alzheimer’s patients, and vomiting side effects were much lower than the donepezil group (Akhondzadeh et al., 2010). Furthermore, these authors also investigated the effects of saffron on mild to moderate Alzheimer’s patients in a placebo-controlled trial to confirm the effects of saffron when compared to placebo. The double-blinded randomized clinical trial compared saffron with memantine and showed almost the same effects and no side effects (Farokhnia et al., 2014).

Furthermore, combined saffron and Kamiuntanto—or Kamiuntantokabankoka in Kampo terminology—should be used to limit dementia.

## Conclusions and Perspectives

Kamiuntanto activity against dementia was determined by *in vitro* and *in vivo* model systems. Based on these findings, the major Kamiuntanto constituents were assayed for their contribution to these activities. For the *Polygala* radix drug, onjisaponin (Figure 1) and sinapinic acid were identified as major chemical contributors to Kampo activity. Furthermore, numerous anti-dementia related activities of the *P*. *ginseng* root drug in Kamiuntanto were also supported by experimental evidence. Thus, both drugs may play important synergistic roles in Kamiuntanto formulas.

For 3,500 years, saffron has not only been used as a medical drug but also as a food spice, and is “Generally Recognized as Safe” by the American Food And Drug Administration (FDA) (Department Of Health And Human Services; Subchapter B—Food For Human Consumption (Continued); Part 182—Substances Generally Recognized as Safe/https://www.accessdata.fda.gov/scripts/cdrh/cfdocs/cfcfr/cfrsearch.cfm?fr=182.20).

Crocin increased intracellular glutathione levels and prevented cell death in PC-12 cells cultured in DMEM-in a brain ischemia model. In these cells, reactive oxygen species generation activated neural sphingomyelinase resulting in ceramide production, which induced cell death as ceramide-release activates the caspase system. However, glutathione directly inhibited neural sphingomyelinase activation. We hypothesize that crocin may prevent neural sphingomyelinase activation in PC-12 cells cultured in DMEM- *via* a GSH-dependent inhibition mechanism.

As indicated, crocin is a major active constituent of saffron which improves learning and memory and prevents LTP blockade by ethanol in an *in vivo* mouse model. However, the oral administrations of saffron and crocin had no effect on memory acquisition in control mice. Naghibi et al. investigated the effects of saffron extracts on morphine-induced memory impairment and concluded saffron extracts attenuated this impairment (Naghibi et al., 2012). We also demonstrated, for the first time, that crocin selectively antagonized the inhibitory effects of ethanol on NMDA-receptor-mediated responses in hippocampal neurons (Abe et al., 1998). We observed that the efficacy of individual crocetin glycosides toward the inhibition of LTP blocking activity by ethanol was directly proportional to the number of sugar moieties in the respective molecules with crocin—containing four glucose moieties—exhibiting the strongest overall effect (Abe et al., 1998). Interestingly, increased bioactivity levels proportional to the number of sugar moieties in a series of related glycosides were previously reported for several other natural product classes, such as cardiac steroids (Shimada et al., 1986), streptozotocin (Gunnarsson et al., 1974), ginsenosides (Takemoto et al., 1984), saikosaponins (Abe et al., 1978), and hemolytic saponins (Voutquenne et al., 2002).

Currently in Japan, 25% of the population has reported occasional sleeping problems for which saffron could be prescribed in combination with Kampo. Saffron has been tested as a sleep promoter and increased total time for non-REM sleep. Thus, the accumulated evidence suggests the clinical benefit of saffron. The indirect relationship between saffron and/or crocin and dementia, *via* sleeping or depression disorders, was characterized. Furthermore, the evidence suggests that saffron improves Alzheimer’s disease symptoms in clinical trials where market medicines were used as controls. Importantly, in 2006, crocin was approved by the Chinese State FDA for clinical trials and became an officially registered drug for angina.

## Abbreviations

DMEM, Dulbecco’s modified Eagle’s medium; FB1, Fumonisin B1; c-GCS, c-Glutamylcysteinyl synthase; GPx, Glutathione peroxidase; GR, Glutathione reductase; GSH, Glutathione; JNK, c-Jun kinase; LTP, Long-term potentiation; NGF, Nerve growth factor; NMDA, N-methyl-D-aspartate; PS, Phosphatidylserine; SAPK, Stress-activated protein kinase; SM, Sphingomyelin.
